# Association Between Family Meal Frequency and Social and Emotional Skills Among Chinese Adolescents: Evidence from the China Family Panel Studies (CFPS)

**DOI:** 10.3390/bs16071098

**Published:** 2026-07-02

**Authors:** Xiaoqing Hu, Zhengyang Wang, Haiping Xue, Nan Guo

**Affiliations:** 1 College of Education, Capital Normal University, Beijing 100048, China; huxq@cnu.edu.cn; 2 College of Preschool Education, Beijing Institute of Education, Beijing 100120, China; wangzhengyang@bjie.ac.cn; 3 Teachers’ College, Beijing Union University, Beijing 100011, China

**Keywords:** family meal frequency, social and emotional skills, adolescents, family socialization, socioeconomic status

## Abstract

Adolescents’ social development is shaped by everyday family interactions, yet evidence on whether routine family practices are associated with social and emotional skills remains limited, particularly in China. Using data from the 2016–2022 China Family Panel Studies, this study examined the association between family meal frequency and five CFPS-based proxy indicators of social and emotional skills among 3727 adolescent-wave observations from adolescents aged 10–16 years. Ordinary least squares models were estimated, with propensity score matching used as a robustness check and subgroup analyses supplemented by formal interaction tests by school stage, residence, and family socioeconomic status (SES). More frequent family meals were positively associated with the proxy indicators for agreeableness, extraversion, and emotional stability, while conscientiousness showed weaker but positive evidence of association; no significant association was found for openness. The subgroup and interaction analyses suggested some group-specific patterns, including tentative differences by school stage and somewhat broader positive associations among rural and low-SES adolescents, although the interaction evidence was limited. The findings suggest that regular family meals may represent a routine family interaction context modestly associated with selected proxy indicators of adolescents’ social and emotional skills.

## 1. Introduction

Adolescence is a formative period for the development of social and emotional skills (Silvers, 2022). These skills shape how young people regulate emotions, build relationships, and manage everyday challenges (Domitrovich et al., 2017; Eriksen & Bru, 2023). They are also important for psychosocial adjustment and later outcomes including educational attainment, social functioning, and well-being (Gómez-López et al., 2022; Israel et al., 2022; Smithers et al., 2018). In the OECD framework, social and emotional skills are organized into five broad domains that are aligned with the Big Five taxonomy: Conscientiousness, openness, extraversion, agreeableness, and emotional stability (OECD, 2015, 2021). In this study, these domains are treated as Big Five-aligned social and emotional skill dimensions rather than as direct measures of personality traits. Recent research has increasingly emphasized the importance of social, emotional, and behavioral skills for adolescents’ adjustment and success in school and beyond (Algan & Huillery, 2025; Huttunen et al., 2024; Soto et al., 2024). Understanding the family and social conditions associated with these skills is therefore important for supporting adolescents’ growth and development.

Families play a central role in adolescents’ social and emotional development. Beyond providing material resources, families may shape development through everyday interactions, emotional support, and behavioral guidance. Research has long emphasized the importance of family relationships and family contexts in shaping adolescents’ psychosocial and social development (Dmitrieva et al., 2004; Morris et al., 2007; Schulz et al., 2023). Emerging evidence also suggests that regular family routines may help strengthen parent–adolescent relationships and provide a stable context for development (Boat et al., 2024). Because such practices are embedded in daily life, they may be relevant to adolescents’ social and emotional skills. Although the benefits of school-based social and emotional learning programs are well documented (Taylor et al., 2017), much less is known about how everyday family practices are associated with these skills.

An ecological perspective provides a useful basis for understanding why family meal frequency may be associated with adolescents’ social and emotional skills. From this perspective, adolescents’ development is shaped not only by broader social conditions but also by repeated experiences in their immediate everyday contexts. The family is one of the most immediate microsystems in which such experiences occur (Bronfenbrenner & Morris, 2006; Morris et al., 2007). Family meals can therefore be viewed as a routine setting within this family microsystem (Fiese & Schwartz, 2008; Fiese et al., 2002). Their relevance lies not in eating together per se, but in the recurring opportunities they may provide for parents and adolescents to spend time together, communicate, express and respond to emotions, model social behavior, and convey everyday norms (Elgar et al., 2013; Fiese & Schwartz, 2008). At the same time, meal frequency cannot capture the quality of mealtime interaction. Previous research has shown that the meaning of family meals may vary according to mealtime structure, emotional climate, communication quality, and broader family functioning (McCullough et al., 2016; Smith et al., 2022; Snuggs & Harvey, 2023). Thus, in the present study, family meal frequency is interpreted as an indicator of repeated opportunities for shared family time rather than as direct evidence of supportive communication or positive family functioning.

Within this ecological framework, different social and emotional skill domains may be linked to different aspects of these repeated family experiences. Opportunities for everyday communication and social interaction may be relevant to the interpersonal and prosocial aspects of agreeableness, particularly those involving empathy, perspective taking, and supportive relationships (Boele et al., 2019; Van Heel et al., 2020). The regular structure of shared family routines may also be relevant to conscientiousness, given that this domain is closely connected to self-regulation, responsibility, and goal-directed behavior (Barton et al., 2019; Eisenberg et al., 2014; Kim & Kochanska, 2019). Mealtime conversations may offer adolescents opportunities to express themselves and participate in social interaction, which may be relevant to extraversion. Similarly, emotional responses and reassurance during shared family time may be relevant to emotional stability by offering everyday opportunities for emotional regulation and security (Boullion et al., 2023; Ratliff et al., 2023). The link with openness may be less direct. Although shared meals could include conversations that stimulate curiosity and expose adolescents to new ideas, more frequent family meals do not necessarily imply more cognitive stimulation, exploratory discussion, or exposure to diverse experiences (Abu Raya et al., 2023; Silvia & Christensen, 2020). This suggests that the association between family meal frequency and openness may be weaker than associations with other domains, as openness may depend more on the cognitive content of shared family interactions. Despite these insights, several important gaps remain. Existing research on family meals has focused largely on adolescents’ mental health symptoms, subjective well-being, and risk behaviors (Le Moal et al., 2025; Snuggs & Harvey, 2023). Some studies have also linked family meals to outcomes more closely related to social and emotional development, such as parent–adolescent communication, family connectedness, parental monitoring, emotional well-being, and behavioral adjustment (Elgar et al., 2013; Offer, 2013; Snuggs & Harvey, 2023; Utter et al., 2013; Wong et al., 2021). Positive family meal practices and values have also been associated with young people’s self-regulation (de Wit et al., 2015). These findings suggest that family meals may be relevant to relational, emotional, and regulatory processes. However, direct evidence linking family meal frequency to multidimensional social and emotional skill domains remains limited. This is an important omission because social and emotional skills reflect broader capacities related to relationship building, emotional regulation, and goal-directed behavior (Domitrovich et al., 2017; Soto et al., 2024). More focused evidence is therefore needed to clarify whether family meal frequency is associated with key dimensions of adolescents’ social and emotional skills.

The evidence base is also geographically narrow. Most existing studies have been conducted in Western settings, with much less known about Chinese adolescents. This matters because, in China, shared meals often carry meanings that extend beyond eating (Oxfeld, 2017; Yu et al., 2015). In many Chinese families, eating together is closely connected to family cohesion, everyday care, and intergenerational relationships. These meanings are consistent with family-oriented cultural values that emphasize relational harmony and mutual obligation (Ban et al., 2025). Against this background, family meals may be a meaningful routine in the daily lives of Chinese adolescents (Boat et al., 2024). This may be particularly relevant because Chinese adolescents often face strong academic expectations and examination-related pressure. When schoolwork occupies much of daily life, shared meals may become one of the regular occasions for family communication, emotional support, and everyday guidance (Kulakow et al., 2021; Sun et al., 2013). Further research in the Chinese context is therefore needed to examine whether family meal frequency is associated with adolescents’ social and emotional skills.

Methodological limitations further constrain the literature. Much of the existing research relies on correlational designs, making it difficult to rule out selection bias arising from individual characteristics, family resources, and other confounding factors (Le Moal et al., 2025; Snuggs & Harvey, 2023). As a result, observed associations between family meals and adolescent developmental outcomes may partly reflect underlying differences in adolescents and family circumstances, rather than family meal frequency itself. More rigorous analyses are therefore needed to assess whether the association between family meal frequency and adolescents’ social and emotional skills holds after accounting for observable differences between families.

In addition, even if an overall association is observed, it may differ across adolescents. The meaning and role of family meals are likely to vary by adolescents’ developmental stage and family circumstances. Adolescents at different school stages face distinct developmental demands, parent–child relationships, and academic pressures, which may shape how family meal frequency relates to specific dimensions of social and emotional skills (Kulakow et al., 2021; Steinberg & Morris, 2001; Van Doorn et al., 2011). Empirical studies also suggest that family meal patterns vary by age or grade level, socioeconomic background, family structure, parental employment, and urban–rural location (López-Gil et al., 2025; McCullough et al., 2016; Neumark-Sztainer et al., 2013; Snuggs & Harvey, 2023). Prior research has examined whether associations between family meals and adolescent outcomes differ by grade level, family affluence, or other sociodemographic characteristics, although evidence on moderation remains mixed (Elgar et al., 2013; Snuggs & Harvey, 2023). Beyond differences in meal patterns, adolescents’ access to structured developmental opportunities outside the home, such as extracurricular activities and private supplementary education, may also vary by family socioeconomic background and residence (Li & Qiu, 2018; Pan et al., 2022; J. Wang & Wu, 2023). In families with fewer resources, family meals may therefore be more salient as a routine source of support and connection (Fielding-Singh, 2017). These differences suggest that it is useful to examine whether this association varies by school stage, residence, and family socioeconomic status.

Against this background, this study uses data from the 2016–2022 waves of the China Family Panel Studies (CFPS) to examine the association between family meal frequency and adolescents’ social and emotional skills. Ordinary least squares (OLS) models and propensity score matching (PSM) are employed to estimate the association and assess the robustness of the findings. The study also explores whether this association varies by school stage, family residence, and family socioeconomic status. In doing so, it provides empirical evidence from China on the association between a routine family practice and adolescents’ social and emotional development. Based on the above, and given that the outcome variables are operationalized as CFPS-based proxy indicators of adolescents’ social and emotional skill domains, the following hypotheses are proposed:

**Hypothesis 1a.** 
*Family meal frequency is positively associated with the proxy indicator of agreeableness among adolescents.*

**Hypothesis 1b.** 
*Family meal frequency is positively associated with the proxy indicator of conscientiousness among adolescents.*

**Hypothesis 1c.** 
*Family meal frequency is positively associated with the proxy indicator of openness among adolescents.*

**Hypothesis 1d.** 
*Family meal frequency is positively associated with the proxy indicator of extraversion among adolescents.*

**Hypothesis 1e.** 
*Family meal frequency is positively associated with the proxy indicator of emotional stability among adolescents.*

**Hypothesis 2.** 
*The association between family meal frequency and adolescents’ social and emotional skills varies by school stage, residence, and family socioeconomic status.*

## 2. Materials and Methods

### 2.1. Study Design and Participants

This study draws on publicly available data from the 2016, 2018, 2020, and 2022 waves of the China Family Panel Studies (CFPS). The CFPS is a large, nationally representative social survey in China that covers 25 provinces, autonomous regions, and municipalities. It uses multistage probability sampling with implicit stratification and probability-proportional-to-size (PPS) procedures. Data collection was approved by the Peking University Biomedical Ethics Committee, and written informed consent was obtained from all participants or their legal guardians.

Data from the 2016, 2018, 2020, and 2022 waves were pooled to form a multi-wave analytic sample. These waves were selected because 2022 is the most recent publicly available CFPS wave, while pooling data across waves allowed us to maximize sample size. Because the study pooled multiple waves of data, the unit of analysis was the adolescent-wave observation rather than the unique adolescent. Some adolescents contributed observations in more than one survey wave. The sample was derived in three steps. First, adolescents aged 10–16 years were retained (*n* = 4380). Second, because family meal frequency was assessed in the context of shared meals with family members, adolescents who were not living with their families were excluded, leaving 3875 adolescent-wave observations. Third, observations with missing or invalid responses on the family meal variable were removed. This resulted in a final pooled sample of 3727 adolescent-wave observations from 2726 unique adolescents. Among these adolescents, 1884 contributed one observation, 683 contributed two observations, and 159 contributed three observations.

For variables other than family meal frequency, models were estimated using complete cases. Specifically, each model retained observations with complete data on the dependent variable, family meal frequency, and the covariates included in that model. Because some social and emotional skill-related proxy indicators were not available in every survey wave or questionnaire module, the analytic sample size varied across outcome models. The extent of outcome missingness and the complete-case analytic sample size for each outcome model are reported in Table S1.

### 2.2. Measures

#### 2.2.1. Dependent Variable

The dependent variables were five CFPS-based proxy indicators of adolescents’ social and emotional skills. Because the CFPS does not include a standardized instrument specifically designed to assess social and emotional skills, available items were used to construct conceptually related indicators based on the OECD five-domain framework, which is aligned with the Big Five taxonomy (Chernyshenko et al., 2018). Brief scales and proxy measures are commonly used in large-scale surveys when comprehensive psychosocial assessments are not available (Gosling et al., 2003; Lang et al., 2011). Accordingly, these measures should be interpreted as Big Five-aligned proxy indicators of the corresponding social and emotional skill domains, rather than as direct measures of standardized social and emotional skills. For consistency with the OECD five-domain framework, the following subsections use the domain labels of agreeableness, conscientiousness, openness, extraversion, and emotional stability. However, these labels should be understood as referring to CFPS-based proxy indicators of the corresponding skill-related domains, rather than to comprehensive measures of each construct. The five proxy indicators were constructed as follows.

(1) Agreeableness: Because the CFPS does not include a standardized agreeableness scale, agreeableness was approximated using a single item reflecting adolescents’ interpersonal relationships. Respondents were asked to rate their interpersonal relationships on a scale from 0 to 10. This item has been used in prior CFPS-based studies and was retained here because it captures one interpersonal and relational aspect that is conceptually relevant to agreeableness (Allen et al., 2022; Huang et al., 2024). However, we acknowledge that this single item cannot capture the full breadth of agreeableness, which includes broader tendencies such as cooperation, empathy, trust, and prosocial orientation. Therefore, this variable should be interpreted as an agreeableness-related interpersonal relationship proxy rather than as a comprehensive measure of agreeableness. Higher scores indicate more positive interpersonal relationships on this proxy indicator.
(2) Conscientiousness: Conscientiousness was approximated using six items reflecting academic diligence, self-discipline, and responsible learning behaviors, including studying hard and checking homework. Responses were rated on a five-point scale from 1 (“strongly disagree”) to 5 (“strongly agree”). Although these items do not capture all facets of conscientiousness, they are conceptually related to core aspects of the domain, such as effort, responsibility, and self-regulation in learning contexts. Higher total scores indicate higher levels on this conscientiousness-related proxy measure. Internal consistency was acceptable (Cronbach’s alpha = 0.729).
(3) Openness: Consistent with prior CFPS-based research, openness was approximated using five items, including the perceived importance of television and the internet as sources of information (Yang & Song, 2025). Responses were rated on a five-point scale from 1 (“not important at all”) to 5 (“very important”). Given the available CFPS items, this measure is best understood as an openness-related information orientation proxy. It reflects adolescents’ perceived importance of external information sources, which may be conceptually related to the information-seeking and cognitive engagement aspects of openness. However, it does not capture the full breadth of openness to experience, such as imagination, creativity, aesthetic interest, intellectual curiosity, or preference for novelty. Therefore, this variable should be interpreted as an openness-related proxy rather than as a comprehensive measure of openness or a standardized measure of social and emotional skills. Higher total scores indicate greater perceived importance of external information sources on this proxy indicator. Internal consistency was acceptable (Cronbach’s alpha = 0.718), although this reliability estimate does not eliminate the construct validity limitations noted above.
(4) Extraversion: Consistent with prior CFPS-based research, extraversion was approximated using three items, including respondents’ confidence in the future (Guan et al., 2024; Yang & Song, 2025). Because the items were measured on different scales, they were standardized before being combined. Given the content of the available CFPS items, this measure is best understood as an extraversion-related positive orientation proxy. Although positive affect, confidence, and energetic engagement are conceptually related to the broader extraversion domain, these items do not directly capture core facets of extraversion such as sociability, assertiveness, talkativeness, or social expressiveness. Therefore, this variable should be interpreted as an extraversion-related positive orientation proxy rather than as a comprehensive or standardized measure of extraversion. Higher scores indicate higher levels of positive orientation on this extraversion-related proxy indicator. Internal consistency was modest (Cronbach’s alpha = 0.649), suggesting that findings related to this measure should be interpreted with caution.
(5) Emotional Stability: Emotional stability was approximated using the eight-item short form of the Center for Epidemiologic Studies Depression Scale (CES-D8). Example items included “feeling depressed”. Responses were rated on a four-point scale from 1 (“rarely or none of the time”) to 4 (“most of the time”). Negatively worded items were reverse-coded, so that higher scores indicated fewer depressive symptoms and better emotional functioning. Because this measure is based on reverse-coded depressive symptoms, it should be interpreted as an emotional stability-related proxy rather than as a direct trait measure of emotional stability. Higher scores indicate better emotional functioning on this proxy measure of emotional stability. Internal consistency was good (Cronbach’s alpha = 0.748).

Because the five dimensions differed in the number of items and response scales, all scores were standardized to a mean of 0 and a standard deviation of 1 to improve comparability across dimensions.

#### 2.2.2. Independent Variable

The main independent variable was family meal frequency. It was measured using the CFPS question, “In general, on how many evenings per week do you have dinner with your family?” Thus, family meal frequency in this study refers to family dinners. Focusing on dinner helps reduce potential measurement error because breakfast and lunch tend to be less regular. Responses ranged from 0 to 7, with higher values indicating more frequent family dinners.

In the baseline ordinary least squares (OLS) analyses, family meal frequency was treated as a continuous variable. For propensity score matching (PSM), it was recoded as a dummy variable, following prior research on family meals (Fulkerson et al., 2006; Luo et al., 2023). Adolescents who reported family dinner on 5–7 evenings per week were classified as the high-frequency group (coded as 1), whereas those who reported family dinner on 0–4 evenings per week were classified as the low-frequency group (coded as 0). This cut-point was used to distinguish adolescents who had family dinners on most evenings of the week from those with lower family dinner frequency.

#### 2.2.3. Covariates

Covariates included student, family, and school characteristics. Student characteristics included gender, family residence, school stage, only-child status, class rank, and class leader status. Family characteristics focused on family socioeconomic status (SES). Following prior studies, principal component analysis (PCA) was used to construct a continuous SES index based on the highest parental education, occupational status, and parental income. The first principal component was retained as the SES index, with higher scores indicating higher family SES. In the heterogeneity analyses, the sample was divided into high- and low-SES groups using the mean SES score as the cutoff. School characteristics included school location and school quality. Definitions of all variables are provided in Table 1.

Subgroup analyses examined whether the association between family meal frequency and adolescents’ social and emotional skills varied by school stage, family residence, and family socioeconomic status (SES). The sample was stratified into primary school and junior high school students, rural and urban groups, and high- and low-SES groups. Separate models were estimated for each subgroup. Each subgroup model included the same covariates as the baseline specification, except for the grouping variable, and also controlled for survey-wave fixed effects (2016, 2018, 2020, and 2022). To formally assess whether the estimated associations differed across groups, additional models were estimated by including interaction terms between family meal frequency and each grouping variable.

All data cleaning, propensity score matching, and statistical analyses were conducted in Stata 17.0. Unless otherwise noted, all statistical tests were two-sided.

## 3. Results

### 3.1. Sample Characteristics

The analytic sample comprised 3727 adolescent-wave observations. Among them, 1998 were observations from male adolescents (53.61%), 1725 were from junior high school students (46.28%), 1660 were from urban families (44.54%), and 1503 were from only children (40.33%). For PSM, the sample was divided into a low-frequency family meal group (*n* = 752) and a high-frequency family meal group (*n* = 2975). Descriptive statistics for both groups are reported in Table 2.

Before matching, the two groups differed on several characteristics. Compared with the low-frequency group, the high-frequency group was less likely to include junior high school students, more likely to come from urban families, and more likely to have higher family socioeconomic status (SES). The two groups also differed on several proxy indicators of outcomes. The high-frequency group showed significantly higher levels of agreeableness, conscientiousness, extraversion, and emotional stability, whereas openness did not differ significantly. No significant between-group differences were observed for gender, only-child status, class rank, class leader status, school location, or school quality. Because some variables had missing values, the percentages reported for categorical variables in Table 2 were based on the valid sample for each variable.

### 3.2. Ordinary Least Squares Regression Results

Table 3 reports the adjusted OLS results. Because the dependent variables were standardized proxy indicators, the coefficients represent changes in standard-deviation units associated with a one-unit increase in family meal frequency. After adjusting for student, family, and school characteristics and survey-wave fixed effects, family meal frequency was positively associated with the proxy indicators for agreeableness (*β* = 0.022, *p* < 0.05), extraversion (*β* = 0.033, *p* < 0.05), and emotional stability (*β* = 0.026, *p* < 0.05). The estimate for the conscientiousness indicator was positive but only marginally significant (*β* = 0.022, *p* < 0.10), whereas the estimate for the openness indicator was close to zero and not statistically significant (*β* = 0.003, *p* > 0.10). Overall, these results point to modest positive associations between family meal frequency and selected social and emotional skill indicators. The VIF diagnostics did not indicate serious multicollinearity among the predictors, and the detailed results are reported in Table S2. The relatively low adjusted *R*^2^ values further suggest that the models had limited explanatory power.

### 3.3. Propensity Score Matching Results

#### 3.3.1. Covariate Balance After Matching

For the PSM analysis, propensity scores were first estimated using a logistic regression model, and covariate balance was then assessed between the high-frequency and low-frequency family meal groups. Table 4 presents the balance results for radius matching (caliper = 0.01), using the agreeableness sample as an illustrative example. The corresponding balance diagnostics for the conscientiousness, openness, extraversion, and emotional stability samples are reported in Supplementary Tables S3–S6. After matching, the absolute standardized bias for every covariate was below 5%, and none of the post-matching *t*-tests showed a significant difference between the two groups. These results suggest that covariate balance was substantially improved after matching.

**Figure 1:**
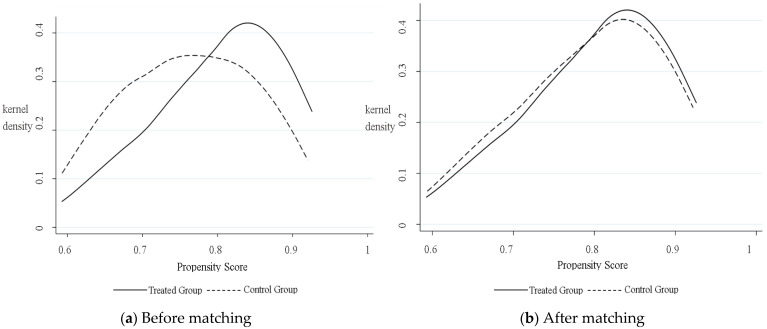
Kernel density of propensity scores before and after matching.

In addition, Figure 1 presents the kernel density distributions of the propensity scores before and after matching. The two groups differed clearly before matching, but their distributions overlapped much more closely after matching, indicating adequate common support. Additional common support diagnostics by outcome model are reported in Table S7.

#### 3.3.2. PSM Estimation Results

Table 5 presents the ATT estimates based on four matching methods: radius matching (caliper = 0.01), 1:1 nearest-neighbor matching, caliper matching (caliper = 0.05), and kernel matching. The pattern of results was broadly consistent across specifications. Family meal frequency was positively associated with the proxy indicators for agreeableness, extraversion, and emotional stability under all four matching methods. The estimates for the conscientiousness indicator were also positive across the matched specifications, although several reached only the marginal significance level. In contrast, the openness indicator was not statistically significant under any matching method. Overall, the PSM estimates were broadly consistent with the adjusted OLS results and suggested modest to moderate differences in selected proxy indicators between the high- and low-frequency family meal groups.

### 3.4. Heterogeneity Analysis

Table 6 reports the subgroup OLS results and formal interaction tests by school stage, family residence, and family socioeconomic status (SES), using the same covariate specification as in the baseline models. As the outcomes were standardized, coefficients are expressed in standard-deviation units.

By school stage, family meal frequency was positively associated with extraversion (*β* = 0.040, *p* < 0.05) and emotional stability (*β* = 0.039, *p* < 0.01) among primary school students, and with agreeableness (*β* = 0.020, *p* < 0.05) and conscientiousness (*β* = 0.028, *p* < 0.05) among junior high school students. The interaction terms were marginally significant for conscientiousness (*β* = 0.036, *p* < 0.10), extraversion (*β* = −0.040, *p* < 0.10), and emotional stability (*β* = −0.030, *p* < 0.10), suggesting possible differences between the two school stages. No significant interaction was found for agreeableness or openness.

By residence, family meal frequency was positively associated with extraversion among urban adolescents (*β* = 0.025, *p* < 0.05). Among rural adolescents, it was associated with conscientiousness (*β* = 0.028, *p* < 0.05), while the estimate for emotional stability was positive but only marginally significant (*β* = 0.022, *p* < 0.10). The interaction terms for conscientiousness (*β* = −0.027, *p* < 0.10) and emotional stability (*β* = −0.021, *p* < 0.10) were negative and marginally significant, suggesting that the corresponding associations may be more apparent among rural adolescents. No significant interaction was found for agreeableness, openness, or extraversion.

By family SES, family meal frequency was positively associated with agreeableness among adolescents from low-SES families (*β* = 0.034, *p* < 0.01). The estimates for conscientiousness (*β* = 0.028, *p* < 0.10) and emotional stability (*β* = 0.024, *p* < 0.10) in this group were positive but only marginally significant. In the high-SES group, the association with extraversion was marginally significant (*β* = 0.024, *p* < 0.10). The interaction terms for agreeableness (*β* = −0.034, *p* < 0.10) and conscientiousness (*β* = −0.017, *p* < 0.10) were negative and marginally significant, suggesting that these associations may be more apparent among adolescents from low-SES families. The remaining interaction terms were not statistically significant.

Overall, the subgroup results suggest some variation in the associations across groups, with positive associations appearing across a broader range of dimensions among rural adolescents and those from low-SES families. However, the estimated coefficients were small in magnitude, and most significant interaction terms were only marginally significant. Thus, these findings should be viewed as modest subgroup patterns rather than strong evidence of substantial between-group differences.

## 4. Discussion

Social and emotional skills are central to adolescents’ adjustment and development. Although family processes have been widely studied in relation to adolescent development, less is known about whether family meals are associated with adolescents’ social and emotional skills, particularly outside Western settings. In the Chinese context, this study examined this association and its variation across groups. Family meal frequency was positively associated with several CFPS-based proxy indicators of adolescents’ social and emotional skills, which were used to approximate selected domains of adolescents’ social and emotional skills. The subgroup analyses, together with the interaction tests, suggested some group-specific patterns, although the evidence for between-group differences was limited. Positive associations appeared more broadly among adolescents in rural areas and those from low-SES families. Taken together, these findings provide new evidence from China and suggest the potential relevance of everyday family interaction, while also indicating that the observed associations were modest and should be interpreted in light of the relatively low explained variance.

### 4.1. Overall Association Between Family Meal Frequency and Adolescents’ Social and Emotional Skills

The findings suggest that more frequent family meals were positively associated with the proxy indicators for agreeableness, extraversion, and emotional stability, while conscientiousness showed weaker but positive evidence of association. This pattern is broadly consistent with earlier research linking family meals to adolescents’ adjustment and behavioral development (Chen et al., 2024; Rahal et al., 2025). One plausible explanation is that family meals may provide a regular setting for communication, support, and guidance, while also potentially reinforcing everyday expectations about behavior and responsibility (Fiese et al., 2006; Zvara et al., 2026). Over time, these repeated interactions could be related to adolescents’ emotion regulation, social confidence, and self-regulatory and interpersonal capacities (Boat et al., 2024; de Wit et al., 2015; Ochs & Shohet, 2006). These associations may also be related to unmeasured aspects of broader family functioning, such as family cohesion, parental engagement, communication quality, parental monitoring, and household stability, which may partially explain the observed associations. Because these interactional processes were not directly assessed, they should be viewed as possible explanations rather than mechanisms tested in the present study. Overall, family meals may be understood as one everyday family context that is modestly associated with selected social and emotional skill indicators.

The consistently non-significant association with openness may suggest that this indicator is less closely related to routine family meal frequency than to experiences involving novelty, exploration, and exposure to diverse ideas, such as reading, extracurricular activities, peer interaction, or broader cultural resources (Kaufman et al., 2016; Schwaba et al., 2018; W. Wang et al., 2024). Family meals may be more directly connected to interpersonal and emotional aspects of development, which may help explain why associations were more evident for agreeableness, extraversion, and emotional stability.

### 4.2. Heterogeneity in the Association Between Family Meal Frequency and Adolescents’ Social and Emotional Skills

The subgroup and interaction analyses suggested some variation in the association between family meal frequency and adolescents’ social and emotional skill indicators. Across school stages, more frequent family meals were linked to extraversion and emotional stability among primary school students, but to agreeableness and conscientiousness among junior high school students. The formal interaction tests provided tentative evidence of stage differences for conscientiousness, extraversion, and emotional stability, although these interaction terms were only marginally significant. One possible explanation is that family meals may be associated with different developmental needs at different stages (Chaku & Davis-Kean, 2024). For younger adolescents, regular family meals may primarily serve as a stable setting for emotional support and everyday interaction, which may be especially relevant to social confidence and emotional regulation (De Raeymaecker & Dhar, 2022; Silvers, 2022). For junior high school students, the same routine may matter more as a setting in which behavioral expectations, responsibility, and self-control are reinforced, which may be more closely tied to agreeableness and conscientiousness at this stage (Farley & Kim-Spoon, 2014; Tackman et al., 2017). This may help explain why the pattern of association differed across school stages.

The results also showed that positive associations were observed across a wider range of dimensions among adolescents in rural areas and those from low-SES families. The interaction tests provided some, although limited, support for these subgroup patterns. When developmental resources outside the home are more limited, stable family routines and everyday family interaction may become more salient (Ayoub et al., 2018; Bernardi, 2014; Ng-Knight & Schoon, 2017). By contrast, adolescents from urban or high-SES families often have access to a wider range of developmental opportunities beyond the family, including organized extracurricular activities and other structured forms of enrichment (Li & Qiu, 2018; Weininger et al., 2015). The findings therefore raise the possibility that family meals may be more closely associated with selected social and emotional skill indicators in less advantaged family environments.

### 4.3. Strengths and Limitations

This study has several strengths. Methodologically, the study combines baseline OLS models with propensity score matching (PSM) as a robustness check. This helps improve comparability between the high- and low-frequency family meal groups and strengthens confidence in the main findings. In addition, formal interaction tests were added to complement the subgroup analyses, allowing the study to assess whether the associations differed across school stage, family residence, and family socioeconomic status more directly. It also contributes new evidence from China, a non-Western context shaped by collectivist family norms and relatively high academic pressure, to a literature that has been dominated by Western samples. The subgroup and interaction analyses further suggest some group-specific patterns in the association between family meal frequency and adolescents’ social and emotional skill indicators, although the evidence for between-group differences should be interpreted cautiously. These findings help extend understanding of how everyday family practices are associated with adolescent development across different social contexts and may have relevance for family-based support strategies, particularly in less advantaged contexts.

Several limitations should also be noted. First, the analysis was based on a pooled multi-wave observational sample from the available CFPS waves. Although survey-wave fixed effects were included and PSM was used to reduce selection bias related to observed characteristics, unobserved confounding and omitted-variable bias cannot be ruled out, and causal claims cannot be made. In particular, family meal frequency may partly capture broader family functioning, including family cohesion, parental engagement, and communication quality, rather than reflecting an independent developmental influence of meal frequency alone. Reverse causality is also possible, as adolescents with stronger social and emotional skills may be more likely to participate in family meals or maintain positive family relationships. In addition, the current analysis focused on linear associations and did not examine possible nonlinear or threshold patterns in the relationship between family meal frequency and the outcomes. Sensitivity analyses beyond PSM were also limited; future research could consider fixed-effects approaches, inverse probability weighting, or alternative cutoffs for family meal frequency. Future research could examine these relationships using longitudinal, quasi-experimental, or experimental designs. Second, the measures of social and emotional skills were constructed from available CFPS items using the OECD framework and should therefore be understood as proxy measures rather than standardized instruments. Because some dimensions were measured with single-item or indirect indicators, these measures may not fully capture the breadth of the target constructs. Accordingly, the results should be interpreted as reflecting associations with CFPS-based indicators of these constructs, rather than with fully validated measures of social and emotional skills. Future studies could further test these findings using standardized and multidimensional assessments. Third, the key variables relied largely on adolescent self-reports, and the analysis focused primarily on the frequency of family meals. However, meal frequency should not be equated with the quality of mealtime interaction. It was therefore not possible to examine meal quality or the content of family communication in greater detail. Future research could benefit from multi-informant data and from a closer examination of how the quality of family meals and family interaction relate to adolescent development. Fourth, the complete-case analytic samples differed across outcome models because of outcome-specific missingness and missing values in covariates. As a result, the included observations and excluded observations may differ systematically in ways that could affect the estimates. In addition, CFPS survey weights were not applied in the present analyses, so the findings should be interpreted as associations within the analytic sample rather than as population-level estimates. Future studies using nationally representative weighting procedures may obtain somewhat different estimates.

## 5. Conclusions

This study found that family meal frequency was positively associated with several CFPS-based proxy indicators of social and emotional skills among Chinese adolescents. In the main analyses, positive associations were observed for the proxy indicators of agreeableness, extraversion, and emotional stability, while the association with the conscientiousness indicator was weaker and the openness indicator was not consistently related to family meal frequency. The subgroup analyses and interaction tests suggested some group-specific patterns, with associations appearing somewhat more broadly among adolescents in rural areas and those from low-SES families. However, these subgroup patterns should be interpreted cautiously, as the interaction evidence was limited and most significant interaction terms were only marginally significant. Overall, family meal frequency should be understood as modestly associated with selected CFPS-based proxy indicators, rather than as a major determinant of adolescents’ social and emotional development.

These findings suggest that family meals may be viewed as one accessible and potentially beneficial routine for everyday family interaction. For families, the findings point to the value of creating regular, supportive, and low-conflict mealtime interactions when feasible. For educators and practitioners, family meals may serve as a concrete example in family education or parent guidance programs to encourage realistic routines that create opportunities for parent–child communication. More broadly, the study highlights the potential relevance of everyday family processes for selected CFPS-based proxy indicators of adolescents’ social and emotional skills, although these implications should be considered in light of the observational design and the modest size of the observed associations.

## Figures and Tables

**Table 1:** Study variables.

Type	Variable	Description and Coding
Dependent variables	Agreeableness	Continuous proxy indicator approximated using a single item assessing the quality of interpersonal relationships.
	Conscientiousness	Continuous proxy indicator constructed by summing six items, including studying hard and checking homework.
	Openness	Continuous proxy indicator constructed by summing five items, including the perceived importance of television and the internet.
	Extraversion	Continuous proxy indicator constructed by summing three standardized items, including confidence in one’s future.
	Emotional Stability	Continuous proxy indicator constructed by summing reverse-coded CES-D8 items.
Independent variable	Family meal frequency	The number of evenings per week the adolescent ate dinner with family members. Treated as a continuous variable in the OLS analyses (0–7 days per week) and as a binary indicator in the PSM analyses: low frequency (0–4 days = 0) and high frequency (5–7 days = 1).
Covariates	Gender	0 = Female, 1 = Male
	Family residence	0 = Rural, 1 = Urban
	School stage	0 = Primary school, 1 = Junior high school
	Only-child status	0 = No, 1 = Yes
	Class rank	Rank category in the most recent major examination: 1 = Bottom 24%, 2 = 51–75%, 3 = 26–50%, 4 = 11–25%, 5 = Top 10%
	Class leader status	0 = No, 1 = Yes
	Family socioeconomic status (SES)	Continuous PCA-based composite score derived from the highest parental education, the highest parental occupational status, and the highest parental income
	School location	0 = Rural, 1 = Urban
	School quality	0 = Non-demonstration school, 1 = Demonstration school

Notes: All five dependent variables were standardized as z-scores (mean = 0, SD = 1). School quality was proxied by demonstration school status, which is commonly associated with greater resources and a stronger reputation in China.

**Table 2:** Descriptive characteristics of the study sample.

Variables	Total Sample (*n* = 3727)	Family Meal Frequency Group		*p*-Value
		Low Frequency (*n* = 752)	High Frequency (*n* = 2975)	
Agreeableness	0.00 (1.00)	−0.08 (0.95)	0.02 (1.02)	0.023
Conscientiousness	0.12 (0.99)	−0.02 (0.89)	0.16 (1.01)	0.004
Openness	−0.05 (1.05)	−0.05 (1.04)	−0.06 (1.06)	0.870
Extraversion	0.01 (0.99)	−0.10 (0.97)	0.04 (1.00)	0.015
Emotional Stability	0.04 (1.00)	−0.05 (1.00)	0.08 (0.99)	0.002
Male	1998 (53.61%)	404 (53.72%)	1594 (53.58%)	0.940
Junior high school	1725 (46.28%)	472 (62.77%)	1253 (42.12%)	<0.001
Urban residence	1660 (44.54%)	279 (37.10%)	1381 (46.42%)	<0.001
SES	0.01 (1.01)	−0.16 (0.93)	0.05 (1.03)	<0.001
Only-child status	1503 (40.33%)	303 (40.29%)	1200 (40.34%)	0.980
Class rank				0.078
Bottom 24%	196 (6.37%)	39 (5.81%)	157 (6.53%)	
51–75%	332 (10.79%)	70 (10.43%)	262 (10.89%)	
26–50%	800 (26.01%)	200 (29.81%)	600 (24.95%)	
11–25%	875 (28.45%)	193 (28.76%)	682 (28.36%)	
Top 10%	873 (28.38%)	169 (25.19%)	704 (29.27%)	
Class leader status	1232 (34.30%)	256 (35.16%)	976 (34.08%)	0.580
Urban school location	1788 (49.41%)	381 (51.50%)	1407 (48.90%)	0.200
Demonstration school	748 (23.36%)	148 (24.54%)	600 (23.09%)	0.450

Notes: Continuous variables are presented as mean (SD) and categorical variables as *n* (%). Percentages are based on valid cases. Group differences were tested using *t*-tests and chi-square tests, as appropriate.

**Table 3:** Main adjusted OLS regression results.

Variables	Agreeableness	Conscientiousness	Openness	Extraversion	Emotional Stability
Family meal frequency	0.022 ** [0.002, 0.042]	0.022 * [−0.004, 0.049]	0.003 [−0.029, 0.034]	0.033 ** [0.004, 0.062]	0.026 ** [0.002, 0.049]
Male	0.073 * [−0.014, 0.159]	−0.118 ** [−0.226, −0.009]	0.077 [−0.036, 0.190]	0.231 *** [0.106, 0.355]	0.023 [−0.066, 0.112]
Urban residence	0.019 [−0.074, 0.112]	0.125 * [−0.000, 0.249]	0.010 [−0.118, 0.138]	0.090 [−0.045, 0.225]	0.027 [−0.071, 0.125]
Junior high school	−0.095 ** [−0.185, −0.005]	−0.269 *** [−0.395, −0.142]	0.229 *** [0.111, 0.347]	−0.063 [−0.220, 0.093]	−0.134 *** [−0.227, −0.040]
Only-child status	0.066 [−0.024, 0.156]	−0.149 ** [−0.267, −0.031]	−0.054 [−0.176, 0.069]	−0.033 [−0.179, 0.113]	−0.010 [−0.101, 0.080]
Class rank	0.043 ** [0.007, 0.080]	0.203 *** [0.148, 0.258]	−0.018 [−0.072, 0.035]	0.059 ** [0.000, 0.118]	0.102 *** [0.062, 0.142]
Class leader status	0.134 *** [0.043, 0.224]	0.082 [−0.036, 0.200]	−0.029 [−0.148, 0.091]	0.024 [−0.108, 0.155]	0.115 ** [0.025, 0.205]
SES	0.004 [−0.045, 0.052]	−0.035 [−0.096, 0.027]	−0.065 ** [−0.124, −0.005]	−0.066 * [−0.136, 0.004]	0.063 ** [0.014, 0.113]
Urban school location	0.026 [−0.064, 0.115]	0.002 [−0.117, 0.121]	0.079 [−0.046, 0.205]	0.095 [−0.037, 0.227]	0.102 ** [0.007, 0.197]
Demonstration school	0.073 [−0.025, 0.171]	0.155 ** [0.030, 0.279]	0.033 [−0.099, 0.164]	−0.082 [−0.244, 0.081]	−0.132 ** [−0.241, −0.022]
Constant	−0.452 [−1.386, 0.482]	−0.895 *** [−1.300, −0.490]	−0.656 ** [−1.166, −0.147]	−0.095 [−0.670, 0.480]	−0.234 [−0.759, 0.291]
*n*	2111	1172	1207	1018	2130
F statistic	3.83 ***	11.33 ***	3.69 ***	3.16 ***	7.85 ***
Adjusted R-squared	0.016	0.110	0.021	0.024	0.042
Survey-wave fixed effects	Yes	Yes	Yes	Yes	Yes

Notes: Coefficients are reported with 95% confidence intervals in brackets. All dependent variables were standardized as z-scores. Standard errors were clustered at the individual level. * *p* < 0.10, ** *p* < 0.05, *** *p* < 0.01.

**Table 4:** Covariate balance for the agreeableness sample before and after radius matching (caliper = 0.01).

	Matching	Treated Group	Control Group	Standardized Bias (%)	*t* Statistic
Gender	Before	0.547	0.570	−4.5	−0.83
	After	0.549	0.556	−1.5	−0.43
Family residence	Before	0.491	0.338	20.8	3.80 ***
	After	0.487	0.504	−3.4	−0.97
School stage	Before	0.451	0.660	−42.9	−7.78 ***
	After	0.454	0.462	−1.7	−0.48
Only-child status	Before	0.399	0.388	2.2	0.41
	After	0.395	0.397	−0.4	−0.12
Class rank	Before	3.655	3.586	5.9	1.06
	After	3.649	3.665	−1.4	−0.41
Class leader status	Before	0.357	0.362	−1.1	−0.19
	After	0.356	0.350	1.3	0.37
SES	Before	0.078	−0.170	25.6	4.55 ***
	After	0.056	0.067	−1.2	−0.33
School location	Before	0.490	0.494	−0.8	−0.15
	After	0.491	0.493	−0.5	−0.14
School quality	Before	0.232	0.244	−2.5	−0.46
	After	0.235	0.243	−2.0	−0.59

Notes: Treated group: high-frequency family meal group; control group: low-frequency family meal group. *** *p* < 0.01.

**Table 5:** Propensity score matching estimates.

		Radius Matching (Caliper = 0.01)	1:1 Nearest-Neighbor Matching	Caliper Matching (Caliper = 0.05)	Kernel Matching
Agreeableness	ATT	0.153 *** [0.042, 0.264]	0.168 ** [0.034, 0.302]	0.186 *** [0.060, 0.312]	0.139 ** [0.032, 0.246]
Conscientiousness	ATT	0.132 * [−0.021, 0.285]	0.189 * [−0.003, 0.381]	0.148 * [−0.020, 0.316]	0.166 ** [0.017, 0.315]
Openness	ATT	0.059 [−0.122, 0.240]	0.068 [−0.166, 0.302]	0.075 [−0.111, 0.261]	0.049 [−0.117, 0.215]
Extraversion	ATT	0.304 *** [0.146, 0.462]	0.204 ** [0.004, 0.404]	0.285 *** [0.103, 0.467]	0.246 *** [0.092, 0.400]
Emotional stability	ATT	0.203 *** [0.077, 0.329]	0.267 *** [0.095, 0.439]	0.253 *** [0.109, 0.397]	0.197 *** [0.075, 0.319]

Notes: ATT: average treatment effect on the treated. ATT estimates are reported with estimated 95% confidence intervals in brackets. * *p* < 0.10, ** *p* < 0.05, *** *p* < 0.01.

**Table 6:** Subgroup OLS regression results and formal interaction tests.

Subsample/Interaction Term	Agreeableness	Conscientiousness	Openness	Extraversion	Emotional Stability
School Stage					
Primary school	0.008 [−0.017, 0.033]	−0.002 [−0.034, 0.030]	0.005 [−0.035, 0.045]	0.040 ** [0.004, 0.075]	0.039 *** [0.011, 0.068]
Junior high school	0.020 ** [0.001, 0.036]	0.028 ** [0.001, 0.056]	0.001 [−0.024, 0.025]	0.000 [−0.029, 0.029]	0.009 [−0.011, 0.029]
Family meal frequency × Junior high school	0.014 [−0.010, 0.038]	0.036 * [−0.005, 0.077]	−0.006 [−0.053, 0.041]	−0.040 * [−0.084, 0.007]	−0.030 * [−0.064, 0.004]
Residence					
Rural	0.013 [−0.013, 0.039]	0.028 ** [0.001, 0.056]	−0.003 [−0.036, 0.030]	0.017 [−0.020, 0.055]	0.022 * [−0.004, 0.048]
Urban	0.017 [−0.006, 0.041]	0.017 [−0.017, 0.051]	−0.018 [−0.045, 0.009]	0.025 ** [0.004, 0.047]	0.018 [−0.010, 0.047]
Family meal frequency × Urban	−0.009 [−0.042, 0.024]	−0.027 * [−0.055, 0.000]	−0.014 [−0.056, 0.029]	0.002 [−0.044, 0.047]	−0.021 * [−0.047, 0.004]
Family SES					
Low SES	0.034 *** [0.012, 0.058]	0.028 * [−0.000, 0.058]	−0.017 [−0.048, 0.014]	0.020 [−0.011, 0.052]	0.024 * [−0.000, 0.049]
High SES	0.000 [−0.026, 0.027]	0.013 [−0.018, 0.046]	−0.002 [−0.039, 0.034]	0.024 * [−0.000, 0.051]	0.021 [−0.013, 0.055]
Family meal frequency × High SES	−0.034 * [−0.069, 0.001]	−0.017 * [−0.032, 0.001]	0.012 [−0.036, 0.059]	−0.001 [−0.046, 0.044]	−0.001 [−0.036, 0.035]
Other covariates included	Yes	Yes	Yes	Yes	Yes
Survey-wave fixed effects	Yes	Yes	Yes	Yes	Yes

Notes: Coefficients are reported with 95% confidence intervals in brackets. Subgroup estimates were obtained from separate OLS models, and interaction terms from pooled OLS models. All models include covariates and survey-wave fixed effects, with standard errors clustered at the individual level. * *p* < 0.10, ** *p* < 0.05, *** *p* < 0.01.

## Data Availability

The data used in this study are available from the China Family Panel Studies (CFPS) data center. Researchers may register and obtain access to the public-use data through the official CFPS platform. In accordance with the CFPS Data User Agreement, the authors may not distribute the original or modified CFPS data on journal websites or third-party platforms.

## Supplementary Materials

The following supporting information can be downloaded at: https://www.mdpi.com/article/10.3390/bs16071098/s1, Table S1: Missingness and complete-case analytic sample sizes by outcome model; Table S2: Variance inflation factor diagnostics for the OLS models; Table S3: Covariate balance for the conscientiousness sample before and after radius matching (caliper = 0.01); Table S4: Covariate balance for the openness sample before and after radius matching (caliper = 0.01); Table S5: Covariate balance for the extraversion sample before and after radius matching (caliper = 0.01); Table S6: Covariate balance for the emotional stability sample before and after radius matching (caliper = 0.01); Table S7: Common support diagnostics for propensity score matching by outcome model.

## Abbreviations

The following abbreviations are used in this manuscript:

OLS	Ordinary least squares
PSM	Propensity score matching
SES	Socioeconomic status
