# Supplementary material for: Association Between Family Meal Frequency and Social and Emotional Skills Among Chinese Adolescents: Evidence from the China Family Panel Studies (CFPS)
Source: Behav Sci (Basel). 2026 Jul 2;16(7):1098. doi: 10.3390/bs16071098 (PMC13405159; doi:10.3390/bs16071098)
Supplement: Supplementary file 1 [file behavsci-16-01098-s001.zip › behavsci-4354575-supplementary.pdf]

Supplementary Materials

Table S1. Missingness and complete-case analytic sample sizes by outcome model.

Outcome Model	Outcome Variable	Outcome available n	Outcome missing n (%)	Complete -case n	Unique adolescents in complete-case sample	Excluded from complete-case model n (%)
Model 1	Agreeableness	3,566	161(4.32%)	2,111	1,711	1,616 (43.36%)
Model 2	Conscientiousness	1,961	1,766(47.38%)	1,172	1,112	2,555 (68.55%)
Model 3	Openness	2,393	1,334(35.79%)	1,207	1,119	2,520 (67.61%)
Model 4	Extraversion	1,610	2,117(56.80%)	1,018	1,018	2,709 (72.69%)
Model 5	Emotional Stability	3,721	6(0.16%)	2,130	1,726	1,597 (42.85%)

Notes: Outcome labels are abbreviated; all outcomes are CFPS-based proxy indicators. Complete-case n refers to observations with complete data on the outcome, family meal frequency, and model covariates.

Table S2. Variance inflation factor diagnostics for the OLS models.

Outcome model	Outcome variable	Mean VIF	Maximum VIF
Model 1	Agreeableness	1.12	1.33
Model 2	Conscientiousness	1.13	1.35
Model 3	Openness	1.14	1.38
Model 4	Extraversion	1.12	1.29
Model 5	Emotional Stability	1.12	1.33

Notes: VIF diagnostics were calculated for the main explanatory variable and covariates.

Table S3. Covariate balance for the conscientiousness sample before and after radius matching (caliper = 0.01).

	**Matching**	**Treated Group**	**Control Group**	**Standardized Bias (%)**	***t* Statistic**
Gender	Before	0.541	0.545	-0.8	-0.10
	After	0.537	0.520	3.4	0.74
Residence	Before	0.462	0.415	9.5	1.21
	After	0.466	0.449	3.3	0.72
School stage	Before	0.418	0.710	-61.6	-7.73***
	After	0.418	0.418	-0.0	-0.00
Only-child status	Before	0.340	0.360	-4.1	-0.53
	After	0.339	0.362	-4.8	-1.05
Class rank	Before	3.749	3.670	6.8	0.88
	After	3.770	3.779	-0.8	-0.18
Class leader status	Before	0.363	0.340	4.8	0.62
	After	0.365	0.353	2.6	0.57
SES	Before	0.059	-0.142	20.4	2.56**
	After	-0.023	-0.042	2.0	0.45
School location	Before	0.472	0.505	-6.6	-0.84
	After	0.457	0.452	1.1	0.23
School quality	Before	0.244	0.235	2.1	0.27
	After	0.241	0.261	-4.8	-1.02

Notes: Treated group: high-frequency family meal group; control group: low-frequency family meal group. * $p < 0.10$, ** $p < 0.05$, *** $p < 0.01$.

Table S4. Covariate balance for the openness sample before and after radius matching (caliper = 0.01).

	Matching	Treated Group	Control Group	Standardized Bias (%)	*t* Statistic
Gender	Before	0.544	0.589	-9.0	-1.17
	After	0.566	0.556	2.1	0.37
Residence	Before	0.505	0.297	43.5	5.55***
	After	0.439	0.436	0.6	0.11
School stage	Before	0.536	0.751	-46.0	-5.79***
	After	0.614	0.602	2.5	0.42
Only-child status	Before	0.518	0.541	-4.5	-0.60
	After	0.506	0.525	-3.7	-0.65
Class rank	Before	3.665	3.574	7.7	1.00
	After	3.622	3.704	-6.9	-1.20
Class leader status	Before	0.362	0.297	13.9	1.79*
	After	0.318	0.318	-0.0	-0.01
SES	Before	0.071	-0.289	37.8	4.75***
	After	-0.112	-0.142	3.3	0.60
School location	Before	0.437	0.354	17.0	2.21**
	After	0.359	0.397	-7.8	-1.39
School quality	Before	0.241	0.272	-7.1	-0.95
	After	0.215	0.253	-8.6	-1.57

Notes: Treated group: high-frequency family meal group; control group: low-frequency family meal group. * $p < 0.10$, ** $p < 0.05$, *** $p < 0.01$.

Table S5. Covariate balance for the extraversion sample before and after radius matching (caliper = 0.01).

	Matching	Treated Group	Control Group	Standardized Bias (%)	*t* Statistic
Gender	Before	0.552	0.539	2.5	0.35
	After	0.551	0.538	2.6	0.44
Residence	Before	0.465	0.444	4.1	0.56
	After	0.456	0.461	-1.0	-0.17
School stage	Before	0.393	0.610	-44.5	-6.03***
	After	0.398	0.412	-2.9	-0.50
Only-child status	Before	0.261	0.257	0.9	0.12
	After	0.258	0.243	3.5	0.60
Class rank	Before	3.636	3.618	1.5	0.20
	After	3.606	3.684	-6.7	-1.16
Class leader status	Before	0.357	0.435	-16.0	-2.18**
	After	0.360	0.363	-0.7	-0.13
SES	Before	0.046	-0.114	16.4	2.16**
	After	-0.034	-0.000	-3.4	-0.59
School location	Before	0.546	0.602	-11.3	-1.53
	After	0.534	0.532	0.4	0.06
School quality	Before	0.224	0.212	3.0	0.40
	After	0.201	0.227	-6.1	-1.05

Notes: Treated group: high-frequency family meal group; control group: low-frequency family meal group. * $p < 0.10$, ** $p < 0.05$, *** $p < 0.01$.

Table S6. Covariate balance for the emotional stability sample before and after radius matching (caliper = 0.01).

	Matching	Treated Group	Control Group	Standardized Bias (%)	*t* Statistic
Gender	Before	0.547	0.562	-2.9	-0.54
	After	0.548	0.539	1.8	0.53
Residence	Before	0.489	0.385	21.2	3.89***
	After	0.486	0.506	-4.1	-1.17
School stage	Before	0.453	0.660	-42.6	-7.77***
	After	0.455	0.473	-3.6	-1.02
Only-child status	Before	0.397	0.384	2.6	0.48
	After	0.394	0.415	-4.2	-1.22
Class rank	Before	3.659	3.592	5.7	1.05
	After	3.655	3.686	-2.6	-0.78
Class leader status	Before	0.359	0.366	-1.4	-0.26
	After	0.358	0.349	1.9	0.56
SES	Before	0.076	-0.179	26.2	4.69***
	After	0.059	0.073	-1.5	-0.41
School location	Before	0.489	0.492	-0.7	-0.12
	After	0.487	0.489	-0.4	-0.12
School quality	Before	0.234	0.242	-2.0	-0.37
	After	0.234	0.247	-2.9	-0.83

Notes: Treated group: high-frequency family meal group; control group: low-frequency family meal group. * $p < 0.10$, ** $p < 0.05$, *** $p < 0.01$.

Table S7. Common support diagnostics for propensity score matching by outcome model.

Outcome model	Outcome variable	PSM analytic sample n	Treated observations	Control observations	Treated on common support n (%)	Total off-support observations
Model 1	Agreeableness	2,111	1,688	423	1,677 (99.35%)	12
Model 2	Conscientiousness	1,172	972	200	964 (99.18%)	9
Model 3	Openness	1,207	998	209	920 (92.18%)	79
Model 4	Extraversion	1,018	777	241	771 (99.23%)	7
Model 5	Emotional Stability	2,130	1,701	429	1,689 (99.29%)	12

Notes: Treated observations refer to adolescents in the high-frequency family meal group. Common support was assessed based on the estimated propensity scores for each outcome-specific complete-case sample.
